# Applying diffusion-based Markov chain Monte Carlo

**DOI:** 10.1371/journal.pone.0173453

**Published:** 2017-03-16

**Authors:** Radu Herbei, Rajib Paul, L. Mark Berliner

**Affiliations:** 1 The Ohio State University, Department of Statistics, Columbus, OH, United States of America; 2 Western Michigan University, Department of Statistics, Kalamazoo, MI, United States of America; Shanxi University, CHINA

## Abstract

We examine the performance of a strategy for Markov chain Monte Carlo (MCMC) developed by simulating a discrete approximation to a stochastic differential equation (SDE). We refer to the approach as *diffusion MCMC*. A variety of motivations for the approach are reviewed in the context of Bayesian analysis. In particular, implementation of diffusion MCMC is very simple to set-up, even in the presence of nonlinear models and non-conjugate priors. Also, it requires comparatively little problem-specific tuning. We implement the algorithm and assess its performance for both a test case and a glaciological application. Our results demonstrate that in some settings, diffusion MCMC is a faster alternative to a general Metropolis-Hastings algorithm.

## Introduction

The advent of Markov Chain Monte Carlo (MCMC) has led to major advances in the application of Bayesian analysis in complex problems. The idea is simply put: faced with a posterior distribution too complicated to compute or simulate from directly (i.e., we cannot readily obtain the normalizer or denominator appearing in Bayes’ Theorem), one develops a Markov chain whose stationary distribution is known to coincide with the target posterior distribution. One then runs that chain, knowing that eventually realizations from the chain form an approximate dependent sample from the posterior. Those realizations are then used to estimate features of the posterior (i.e., posterior expectations of interesting quantities, predictive densities, etc.) [1–3].

For example, in some settings, nonlinearity and/or nonconjugacy of certain components of a large model render the standard Gibbs Sampler unusable. Metropolis-Hastings algorithms and Gibbs-Metropolis hybrids can be suggested, though these approaches can be taxing and may require substantial tuning.

In response to such difficulties, we explore diffusion based strategies for MCMC analysis. That is, one develops a diffusion (a solution, in the sense of Itô, to a stochastic differential equation) whose stationary distribution is the target posterior, see Chapter 5 of [4]. The key idea is certainly not new. Indeed, Langevin MCMC procedures are often suggested for generating candidate states in Metropolis steps in MCMC. In this article, we suggest diffusion MCMC as a stand-alone algorithm.

In part, our motivation for suggesting diffusion MCMC is its simplicity in terms of set-up. There are no probability calculations to perform, as in Gibbs’ Sampling, nor any need for choosing and updating distributions for generating candidate states. Indeed, the approach is recommended as an “off-the-shelf” strategy that can be readily implemented. However, as indicated below, it is not a panacea. Further, issues such as burn-in, mixing, convergence rates, and output analysis remain challenging.

Consider a Bayesian analysis for an unknown quantity *θ* (after the introduction, we allow vector-valued unknowns) based on observational data **Y**, having conditional density *g*(**y** ∣ *θ*). Let *π*(*θ*) denote our prior distribution for *θ*. We are to obtain the posterior distribution for *θ* based on the fixed observation **Y** = **y**,

$$\begin{array}{c} p\left(\theta \right)\;\overset{\text{def}}{=}\;p\left(\theta |y\right)=C{\left(y\right)}^{-1}g\left(y|\theta \right)\pi \left(\theta \right), \end{array}$$ (1)

where *C*(**y**) is the normalizing constant. Consider a one-dimensional stochastic differential equation (SDE)

$$\begin{array}{c} d\theta \left(t\right)=b\left(\theta \right)dt+\sigma \left(\theta \right)dW\left(t\right),\;\theta \left(0\right)=\theta_{0},\;t\geq 0, \end{array}$$ (2)

where *θ*_0_ is some fixed initial value and the *drift* *b*(⋅) and *diffusion* *σ*(⋅) > 0 are specified functions, such that Eq (2) admits a unique weak solution. The initial state *θ*_0_ is a random variable with specified density *p*(*θ*, 0); and *dW*(*t*) represents *white noise*. Specifically, {*W*(*t*): *t* ≥ 0} is a standard Brownian motion process or a Wiener process. Consider the temporal evolution of the probability density function, *p*(*θ*, *t*), of a solution *θ*(*t*). Under regularity conditions requiring *b* and *σ* to be differentiable and satisfy a Lipschitz condition

$$\begin{array}{c} |b\left(\theta \right)-b\left(\theta^{\prime }\right)|+|\sigma \left(\theta \right)-\sigma \left(\theta^{\prime }\right)|\leq K|\theta -\theta^{\prime }|, \end{array}$$

for some constant *K* and for all *θ*, *θ*′, *p*(*θ*, *t*) is the solution to the ordinary partial differential equation, known as the *Fokker-Planck* or *Kolmogorov Forward* Equation,

$$\begin{array}{c} \frac{\partial p}{\partial t}=0.50\;\frac{\partial^{2}}{\partial \theta^{2}}\left(\sigma^{2}\;p\right)-\frac{\partial }{\partial \theta }\left(b\;p\right), \end{array}$$ (3)

subject to the initial condition *p*(*θ*, 0) and the assumption that *θ*(0) and {*W*(*t*): *t* ≥ 0} are independent.

Our interest is in stationary solutions, i.e., solutions that are functionally independent of time, so the partial derivative with respect to *t* is zero. Setting the right-hand side of Eq (3) equal to zero and integrating the result once w.r.t. *θ*, it suffices to find a stationary density *p*(*θ*) such that

$$\begin{array}{c} 0.50\;\frac{\partial }{\partial \theta }\left(\sigma^{2}\left(\theta \right)\;p\left(\theta \right)\right)=b\left(\theta \right)\;p\left(\theta \right)\;, \end{array}$$ (4)

for all values of *θ* in the parameter space. The general solution of Eq (4) is

$$\begin{array}{c} p\left(\theta \right)={\left(c\sigma^{2}\left(\theta \right)\right)}^{-1}\;\exp\left(\int_{0}^{\theta }\frac{2b(z)}{\sigma^{2}\left(z\right)}dz\right), \end{array}$$

where the constant *c* is a normalizer.

We let *p*(*θ*) be the target posterior Eq (1) for our Bayesian model and find appropriate functions *b*(*θ*) and *σ*(*θ*) such that *p*(⋅) satisfies the Eq (4). That is,

$$\begin{array}{cc} b(\theta ) & =0.50\left(\sigma^{2}{\left(\theta \right)}^{\prime }+\sigma^{2}\left(\theta \right)\frac{p{\left(\theta \right)}^{\prime }}{p(\theta )}\right)=0.5\sigma^{2}\left(\theta \right)\left(\frac{\sigma^{2}{\left(\theta \right)}^{\prime }}{\sigma^{2}\left(\theta \right)}+\frac{p{\left(\theta \right)}^{\prime }}{p(\theta )}\right) \end{array}$$

where the derivatives are taken with respect to *θ*. When *p*(*θ*) = *g*(**y** | *θ*)*π*(*θ*) as in Eq (1), we look for functions *b*(⋅) and *σ*^2^(⋅) satisfying

$$\begin{array}{ccc} b(\theta ) & = & 0.50\sigma^{2}\left(\theta \right)\left(\frac{d}{d\theta }\log\left(g\left(y\;|\;\theta \right)\pi \left(\theta \right)\sigma^{2}\left(\theta \right)\right)\right)\\ & = & 0.50\sigma^{2}\left(\theta \right)\left(\frac{g{\left(y\;|\;\theta \right)}^{\prime }}{g(y\;|\;\theta )}+\frac{\pi {\left(\theta \right)}^{\prime }}{\pi (\theta )}+\frac{\sigma^{2}{\left(\theta \right)}^{\prime }}{\sigma^{2}\left(\theta \right)}\right), \end{array}$$ (5)

Having completed this step, we can simulate the diffusion and proceed as in MCMC. This is typically accomplished by forming a discrete-time approximation to Eq (2), that is, a Markov chain approximation to the continuous-time process. There may be many pairs (*b*(⋅), *σ*^2^(⋅)) that work for a fixed Bayesian model. It is important to note that the core of a *diffusion MCMC* (DMCMC) implementation has been completely described.

In this article we discretize the diffusion Eq (2) using the Euler scheme. This method has been extensively discussed in the literature, see for example [5] for a comprehensive overview and [6–8] for recent developments. The solution of the stochastic differential Eq (2) is approximated using a discrete time Markov chain {*θ*_*m*_}_*m* ≥ 0_,

$$\begin{array}{c} \theta_{m+1}=\theta_{m}+hb\left(\theta_{m}\right)+\sigma \left(\theta_{m}\right)h^{1/2}Z_{m+1},\;m\geq 0 \end{array}$$ (6)

where *θ*_0_ = *θ*(0), *h* > 0 is the discretization step-size, and *Z*_*m*+1_ is a realization from a standard Gaussian distribution. Abusing notation, we use *θ* to denote both the continuous-time defined in Eq (2) and the discrete-time process from Eq (6). It is typical to extend {*θ*_*m*_}_*m* ≥ 0_ to a continuous time process via interpolation; however this step is not necessary for this paper. From a practical perspective, we are interested in the process {*θ*_*m*_}_*m* ≥ 0_. Two critical questions arise:

1. Does the discrete stochastic process converge to a stationary, ergodic distribution?
2. If so, is that stationary distribution “close” enough to the target posterior distribution to justify the use of conventional output analysis to enable approximate Bayesian inference?

Unfortunately, there are situations where for any choice of the time step *h* > 0, the Markov chain described by Eq (6) will behave drastically different than the continuous time version Eq (2), see [9] for a discussion on this issue. Nevertheless, in many cases the ergodic properties of the discretized process {*θ*_*m*_}_*m* ≥ 0_ are similar to those of {*θ*(*t*)}_*t* ≥ 0_. In particular, in [10] the author shows that under regularity conditions, the Euler discretization scheme does have a stationary measure which converges at an appropriate rate to the unique stationary measure of the continuous-time SDE. Furthermore, in [9] the authors provide conditions under which the continuous-time Langevin diffusion (defined below in Eq (7)) as well as the discretized version Eq (6) are geometrically ergodic. In [7], the authors study the asymptotic properties of time averages $\left(1/M\right)\sum_{m=1}^{M}F\left(\theta_{m}\right)$, where *F* is a given function. This statistic is the natural estimate for the expected value *E*(*F*(*θ*)) = ∫*F*(*θ*)*dp*. They use Poisson equations to show that under mild regularity conditions, any stationary measure of the Euler-discretized process Eq (6) will be close to the unique stationary measure of the underlying SDE. Their Theorem 5.1 also shows that the time average estimator is of order *O*(*h* + 1/*M*).

To illustrate *diffusion-MCMC* and indicate its potential value and simplicity, examples are reviewed in the next section. Henceforth, we set *σ* ≡ 1, and then find the function *b*(⋅); this approach often has the tag *Langevin*. That is, we restrict ourselves to diffusion processes of the form

$$\begin{array}{c} d\theta \left(t\right)=\frac{1}{2}\frac{\partial }{\partial \theta }\log p\left(\theta \left(t\right)\right)dt+dW\left(t\right),\;t\geq 0, \end{array}$$ (7)

where *p*(⋅) is the posterior distribution Eq (1). We note that application of Eq (6) yields the corresponding transition distribution as

$$\begin{array}{c} \theta_{m+1}|\theta_{m}∼N\left(\theta_{m}+0.5h\nabla \log p\left(\theta_{m}\right),h\right). \end{array}$$

We emphasize again that our goal is to present the benefits of a procedure that has been present in the literature for over a decade. Due to its wild behavior even in some simple cases, it has received little attention, especially from practitioners and applied scientists. It has been proposed (see for example the MALA algorithm presented in [9]) that an additional Metropolis-Hastings step will correct the explosive behavior. The MALA algorithm has been further studied and extended in [11] and [12]. In both cases the improvement comes with an increase in computational complexity. We stress that our goal is to avoid a Metropolis-Hastings accept-reject step and this work is motivated by recent theoretical advances in this direction, see [7]. We explore the efficiency and applicability of DMCMC to high-dimensional problems arising in a Bayesian framework, **without** performing the Metropolis-Hastings correction step. When classical (or adaptive) MCMC fails (for example, due to computational time restrictions or inability to select good proposals), we show that diffusion MCMC is a viable alternative which requires little input from the user and can be computationally more efficient.

## Motivating examples

The multivariate form of the diffusion Eq (2) is written as

$$\begin{array}{c} d\theta (t)=b(\theta (t))dt+\sigma (\theta (t))dW(t),\;t>0, \end{array}$$ (8)

where {***θ***(*t*): *t* ≥ 0} is a *q*-dimensional stochastic process. The initial state is ***θ***(0) and {**W**(*t*), *t* ≥ 0} is a *q*-dimensional vector whose elements are each independent standard Brownian motions. Except for the first one, the examples below were chosen to be suggestive of realistic problems for which other MCMC methods can be difficult. As in Eq (1), we use *g*(**y** ∣ ***θ***) to denote the likelihood function, where **y** is the observed data and *π*(***θ***) to denote the prior density.

One class of problems in which diffusion MCMC may be useful involve nonlinearity. For example, suppose the likelihood function *g* depends on ***θ*** via a “link” function *k*(⋅), that is *g* = *g*(· ∣ *k*(***θ***)). Nonlinear structures may also arise in hierarchically specified priors. Nonlinearity may make both Gibbs sampling and Metropolis algorithms difficult. However, if the nonlinearity does not disable the required differentiation, diffusion MCMC may be comparatively simple. We remark that in such cases, the drift function *b*(⋅) may be unruly. If necessary, selection of the diffusion coefficient *σ*(⋅) may be used to control *b*(⋅). However, for the balance of the article we restrict to Langevin diffusions (*σ* = 1).

**Example 1**. Assume that for *τ*^2^ known, *Y* | *θ* ∼ *N*(*θ*, *τ*^2^) and *θ* ∼ *N*(*μ*, *η*^2^). Of course, we know that *θ* | *Y* = *y* is normally distributed with easily computed mean and variance (these will appear below). Applying Eq (5) with the choice of *σ*(*θ*) = 1, yields

$$\begin{array}{c} b\left(\theta \right)=0.50\left(\frac{y}{\tau^{2}}+\frac{\mu }{\eta^{2}}-\theta \left(\frac{1}{\tau^{2}}+\frac{1}{\eta^{2}}\right)\right). \end{array}$$

Let *α* = 0.50(*τ*^−2^ *y* + *η*^−2^ *μ*) and *β* = 0.50(*τ*^−2^ + *η*^−2^). The solution to

$$\begin{array}{c} d\theta (t)=(\alpha -\beta \theta (t))dt+dW(t) \end{array}$$

is

$$\begin{array}{c} \theta \left(t\right)=\int_{0}^{t}\alpha e^{-\beta (t-s)}ds+\int_{0}^{t}e^{-\beta (t-s)}dW\left(s\right)+\theta \left(0\right)e^{-\beta t}. \end{array}$$

It follows that

$$E\left(\theta \left(t\right)\right)=\int_{0}^{t}\alpha e^{-\beta \left(t-s\right)}ds+E\left(\theta \left(0\right)\right)e^{-\beta t}$$ (9)

$$=\frac{\alpha }{\beta }\left(1-e^{-\beta t}\right)+E\left(\theta \left(0\right)\right)e^{-\beta t}.$$ (10)

It can be shown that

$$\text{var}\left(\theta \left(t\right)\right)=\int_{0}^{t}e^{-2\beta \left(t-s\right)}ds+\text{var}\left(\theta \left(0\right)e^{-\beta t}\right)$$ (11)

$$={\left(2\beta \right)}^{-1}\left(1-e^{-2\beta t}\right)+\text{var}\left(\theta \left(0\right)\right)e^{-2\beta t}.$$ (12)

Returning to the original parameterization, we conclude that as *t* → ∞,

$$\begin{array}{c} E\left(\theta \left(t\right)\right)\to {\left(\frac{1}{\tau^{2}}+\frac{1}{\eta^{2}}\right)}^{-1}\frac{y}{\tau^{2}}+\frac{\mu }{\eta^{2}} \end{array}$$

and

$$\begin{array}{c} \text{var}\left(\theta \left(t\right)\right)\to {\left(\frac{1}{\tau^{2}}+\frac{1}{\eta^{2}}\right)}^{-1}, \end{array}$$

which are the usual posterior mean and variance for this Bayesian model. If the initial condition *θ*(0) is normally distributed, then for each *t*, *θ*(*t*) is also normally distributed. Note that the convergence rate to the stationary distribution is exponentially fast.

**Example 2**. Diffusion MCMC is useful in combining data from highly different likelihoods. Let ***θ*** = (*θ*_1_, …, *θ_K_*) and assume that *Y*_*ij*_|*θ*_*i*_ ∼ *g*_*i*_(⋅|*θ*_*i*_) where *i* = 1, …, *K* and *j* = 1, …, *r*_*i*_ and all *Y*_*ij*_ are conditionally independent. For example, let *g*_*i*_ be the Gaussian pdf with mean *θ*_*i*_ and variance *τ*^2^, and the prior for *θ*_*i*_ be a Cauchy distribution with median *μ* and scale parameter *A*. For a Langevin setting (i.e. *σ* = 1), the *i*^th^ component of the drift coefficient ∇ log *p*(***θ***) is

$$\begin{array}{c} \frac{\partial }{\partial \theta_{i}}\nabla \log p\left(\theta \right)=-\sum_{j=1}^{r_{i}}\frac{\left(\theta_{i}-Y_{ij}\right)}{\tau^{2}}-\sum_{i=1}^{K}\frac{2(\theta_{i}-\mu )}{A^{2}+{\left(\theta_{i}-\mu \right)}^{2}}. \end{array}$$

Note that conjugacy plays no direct role in this approach, though the presence of the Cauchy distribution makes a Gibbs sampler infeasible. This example is further analyzed in the next Section.

**Example 3. (Mixture Models)** Suppose *Y*_1_, …, *Y*_*n*_ are conditionally independent and identically distributed given ***θ*** according to a finite mixture of *m* probability density functions *g*_*i*_(⋅ | *θ*). For example, assume that the conditional distribution of the data is

$$\begin{array}{c} g\left(y|\theta \right)=\prod_{j=1}^{n}\left(\sum_{i=1}^{m}\alpha_{i}g_{i}\left(y_{j}|\theta \right)\right), \end{array}$$

where **y** = (*y*_1_, …, *y_n_*)′, *α*_*i*_ > 0, *i* = 1, …, *m* and ∑_*i*_ *α*_*i*_ = 1. Diffusion MCMC is easily formulated if the derivatives of the *g*_*i*_ with respect to *θ* are easily available, either via formal calculations or by using symbolic software, such as Mathematica. We note that similar steps can be used to treat mixture priors.

**Example 4**. **(Hierarchical Models)**. In many models *g* and *π* are products of a variety of terms, e.g., for conditionally independent observations, *g* is a product; *π* is often represented as a product of hierarchical components. In such cases, we have that

$$\begin{array}{c} \frac{\partial \log(g\pi )}{\partial \theta_{i}}=\frac{\partial \log(g^{\left(i\right)}\pi^{\left(i\right)})}{\partial \theta_{i}}, \end{array}$$

where the superscripts indicate that only those components of *g* and *π* that explicitly depend on *θ*_*i*_ are involved in the calculation. This parallels the familiar step in computing full conditionals in setting up a Gibbs Sampler. Namely, for each *i*, one computes the distributions

$$\begin{array}{c} \left[\theta_{i}|\text{all}\;\text{other}\;\theta_{j}\right]=\frac{g^{\left(i\right)}\pi^{\left(i\right)}}{\int g^{\left(i\right)}\pi^{\left(i\right)}d\theta_{i}}. \end{array}$$ (13)

Suppose that the Bayesian model takes the form **Y** | *θ*_1_, … *θ_q_* ∼ *g*(**y** | *θ*_1_, … *θ_q_*) and

$$\begin{array}{c} \pi \left(\theta_{1},\ldots \theta_{q}\right)=\pi^{1}\left(\theta_{1}|\theta_{2},\ldots \theta_{q}\right)\pi^{2}\left(\theta_{2}|\theta_{3},\ldots \theta_{q}\right)\cdots \pi^{q}\left(\theta_{q}\right). \end{array}$$

We adapt the notation in Eq (5) as follows: for a function *f*(*θ*_1_, … *θ*_*q*_) define

$$\begin{array}{c} f_{\left(i\right)}=\frac{\partial f}{\partial \theta_{i}},\;i=1,\ldots ,q. \end{array}$$

Hence,

$$\begin{array}{c} \frac{\partial \log(g\pi )}{\partial \theta_{i}}=\frac{g_{\left(i\right)}}{g}+\sum_{j=1}^{i}\frac{\pi_{\left(i\right)}^{j}}{\pi^{j}} \end{array}$$ (14)

We note that Gibbs sampling is useful when the full conditionals Eq (13) are readily obtained and simulated. This typically arises when the full conditionals actually depend on a small subset of the parameters in the conditions. This is not necessary in diffusion MCMC.

## Applications

To provide insight into diffusion MCMC (DMCMC), we present a standard test case and a real-data example. Our goal is to assess the performance of DMCMC, especially in comparison with the current *state-of-the art* adaptive MCMC approach, see [13, 14]. The DMCMC methodology is compared to a multivariate adaptive Metropolis sampler (AM). For the AM algorithm, the proposal distribution at iteration *m* is given by

$$\begin{array}{c} \left(1-\beta \right)N\left(x,{\left(2.38\right)}^{2}\Sigma_{m}/q\right)+\beta N\left(0,{\left(0.1\right)}^{2}I_{q}/q\right) \end{array}$$

where Σ_*m*_ is the current estimate of the covariance matrix of the target distribution and *β* is a small positive constant (we take *β* = 0.05). The AM algorithm is widely accepted as one of the best sampling algorithms, especially for complex target distributions where dependencies among parameters make it difficult to select proposal distributions. We refer the reader to [15] for several comparisons between MCMC algorithms. The scaling factor (2.38)^2^ can also be “adapted”; in this case we refer to the procedure as *adaptive scaling within adaptive MCMC*. However, user input is not eliminated completely as there remain tuning parameters to be specified.

For comparisons, we inspect trace-plots to assess convergence and compare algorithms via their *averaged squared jumping distance*

$$\begin{array}{c} ASJD=E({\left(X_{m}-X_{m-1}\right)}^{2}). \end{array}$$

This quantity is estimated by $\frac{1}{M}\sum_{m=1}^{M}{\left(X_{m}-X_{m-1}\right)}^{2}$ for both AM and DMCMC algorithms. Comparatively large *ASJD* indicates the desirable property of fast mixing.

We also add a computational constraint for our examples. We limit ourselves to relatively short runs of the Markov chains (AM and DMCMC). This can be very dangerous for classical MCMC since one will have difficulty assessing whether the chains have reached stationarity. Our examples will show that the diffusion approach quickly finds regions with high posterior probability and explores them thoroughly.

### Synthetic example

Assume that *Y*_*i*1_, …, *Y*_*ir*_*i*__|*θ*_*i*_, *γ* are an iid sample from a *Gaussian*(*θ*_*i*_, *V*(*γ*)) distribution, where 1 ≤ *i* ≤ 1000 and 1 ≤ *j* ≤ *r*_*i*_. We specify the variance *V*(*γ*) to be

$$\begin{array}{c} V\left(\gamma \right)=\frac{a+b\;e^{\gamma }}{1+e^{\gamma }}\;,\;\;\text{for}\;\gamma \in R, \end{array}$$ (15)

where 0 < *a* < *b* < ∞ are specified constants. The reason behind Eq (15) is twofold: (1) we require that all the parameters of the model be supported on the entire real line, hence a transformation is required for all variances, and (2) we aim for a *Uniform*(*a*, *b*) prior distribution for the variance *V*(*γ*). Certainly, other distributions (such as Gamma or Inverse Gamma) can be considered. Using an inverse transformation, this is equivalent to specifying the prior density for *γ* as

$$\begin{array}{c} f\left(\gamma \right)=\frac{e^{\gamma }}{{\left(1+e^{\gamma }\right)}^{2}}\;,\;\gamma \in R\;. \end{array}$$

We let the sample sizes *r*_*i*_ vary between 5 and 500. For *θ*_1_, *θ*_2_, …, *θ*_500_ we specify independent prior distributions, *θ*_*i*_|*μ*, *A* ∼ *Cauchy*(*μ*, *A*), with density proportional to [1 + ((*θ*_*i*_ − *μ*)/*A*)^2^]^−1^. The parameter *A* is held fixed for this example, although it can be treated similarly to the data variance *V*(*γ*). For the hyperparameter *μ* we specify a *Gaussian*(0, 1) prior distribution. Using the independence assumption, the likelihood function is written as

$$\begin{array}{ccc} g_{y}\left(\theta \right) & \equiv & g_{y}\left(\theta_{1},\ldots ,\theta_{1000},\gamma ,\mu \right)\\ & \propto & \prod_{i=1}^{1000}\left(V{\left(\gamma \right)}^{-r_{i}/2}\right)\exp\left\{-\frac{\sum_{j=1}^{r_{i}}{\left(Y_{ij}-\theta_{i}\right)}^{2}}{2V(\gamma )}\right\}, \end{array}$$ (16)

and the prior density is proportional to

$$\begin{array}{ccc} \pi (\theta ) & \equiv & \pi (\theta_{1},\ldots ,\theta_{1000},\gamma ,\mu )\\ & \propto & \left[\prod_{i=1}^{1000}\frac{1}{1+{\left(\frac{\theta_{i}-\mu }{A}\right)}^{2}}\right]\cdot \frac{e^{\gamma }}{{\left(1+e^{\gamma }\right)}^{2}}\cdot \exp\left\{-\mu^{2}/2\right\}\;. \end{array}$$ (17)

**Selection of the time step**. The selection of “good” time steps for DMDMC is challenging. First, time steps that are too large may result in explosive (transient) processes. In general, the user faces a conundrum: a very small time step typically results in chain whose dynamics are similar to those of the target continuous-time diffusion, but is slowly mixing.

**Fig 1:**
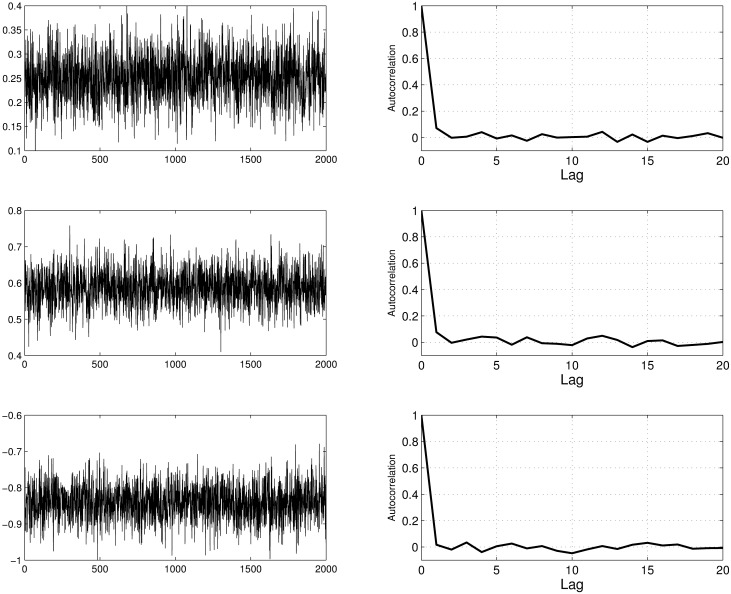
Trace plots and autocorrelation functions for three parameters: *θ*_1_, *θ*_101_, *θ*_201_.

**Fig 2:**
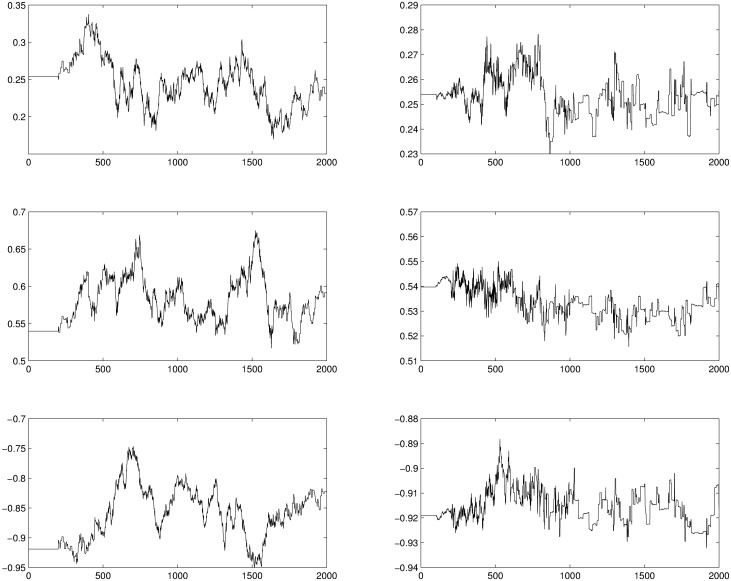
Trace plots for three parameters: *θ*_1_, *θ*_101_, *θ*_201_. The left panels show the results from the adaptive MCMC sampler. The right panels show the *adaptive scaling within adaptive MCMC* sampler.

In our experiments we observed that selecting *h* = *O*(1/*q*) results in a good performance of the DMCMC algorithm for this example. In Fig 1 we display the trace plots and autocorrelation functions for three parameters (results for all parameters where quite similar) from the DMCMC approach. Fig 2 shows trace plots for the same parameters resulting from two adaptive approaches as described above. In each case the chains were run for 20000 iterations and thinned by ten. It is evident from these figures that the adaptive approach has difficulty exploring the state space. The key issue is the dimension of the state space (1000+ in this example). The adaptive approach will not “learn” a one thousand dimensional covariance matrix properly. In Table 1 we summarize the target values and estimates (posterior means) for two parameters, *θ*_1_ and *θ*_201_. Table 2 contains corresponding estimates of ASJD. We see that the estimated ASJD indicates that the mixing rates of the diffusion MCMC algorithm is much higher than the adaptive case.

**Table 1:** Selected DMCMC and AM point estimates.

Parameter	Target value	DMCMC estimate	AM estimate
*θ*_1_	0.2508	0.2517	0.2451
*θ*_201_	-0.9333	-0.8403	-0.8637

**Table 2:** Average squared jumping distance for *θ*_1_ and *θ*_201_.

Parameter	AJSD_*DMCMC*_	ASJD_*AM*_
*θ*_1_	0.0042	0.17 × 10^−4^
*θ*_201_	0.0044	0.16 × 10^−4^

### Glacial dynamics

In [16], the authors present a hierarchical Bayesian analysis for inferring features of the dynamics of the Northeast Ice Stream in Greenland. For our purposes, we use a subset of their data and a simplified version of their model. Glaciers flow under the influence of gravity moderated by resistive forces at its base and sides. For a path roughly along the center of the glacier as it flows toward the sea, physical reasoning and simplifying approximations lead to a model for surface velocity of the stream as a function of ice thickness and the shape of the surface. Let *x* (*m*) denote a spatial location. The model for the surface velocity *u*(*x*) (*ms*^−1^) used here is

$$\begin{array}{c} u\left(x\right)=u_{bx}+\left(0.50\right)\;A\left(x\right)\;{\left(\rho g\right)}^{3}\;H^{4}\left(x\right)\;{\left(\frac{ds(x)}{dx}\right)}^{3}, \end{array}$$ (18)

where *u*_*bx*_ is sliding velocity, *s*(*x*) (*m*) is ice-surface elevation, *H*(*x*) (*m*) is ice thickness, *ρ* = 911 *kgm*^−3^ is the density of ice, and *g* = 9.81 *ms*^−2^ is the gravity constant. Though the quantity *A*(*x*) depends on temperature, it is often treated as a constant *flow parameter*. In this article we model *A* using a Fourier expansion

$$\begin{array}{c} A\left(x\right)=a_{0}+\sum_{k=1}^{3}a_{k}\cos\left(kx\omega \right)+b_{k}\sin\left(kx\omega \right). \end{array}$$ (19)

We assume the following quadratic model for the surface:

$$\begin{array}{c} s\left(x\right)=\beta_{1}x^{2}+\beta_{0}, \end{array}$$ (20)

where *β*_0_ and *β*_1_ are unknown parameters. The authors in [16] use a different, more complicated functional form for *s*. We found Eq (20) sufficient for our purposes. Let *B*(*x*) be the elevation of the base of the glacier so that

$$\begin{array}{c} H(x)=s(x)-B(x). \end{array}$$

The dataset consists of vectors **S** observed at fill-in spatial locations covering approximately 200 *km* of the ice stream, the observed surface topography; **B**, the observed basal topography; and **U**, surface velocities. Additional description of the data is given in [16].

#### Data models

Let ***θ*** represent the set of all parameters introduced in the modeling. We assume that **S**, **B**, **U** are conditionally independent given ***θ***.

**Surface Data**. The data model for **S** is a conventional Gaussian measurement error model:

$$\begin{array}{c} S|\theta ∼N(s,\sigma_{S}^{2}\;I), \end{array}$$ (21)

where **s** is the vector of values of Eq (20) at the observation locations; $\sigma_{S}^{2}$ is an unknown measurement error variance; and *I* is the identity matrix.

**Basal Data**. It is argued (see [16]) that the basal data must be smoothed to be useful in Eq (18). Following their approach we partition the domain of the data into 2^10^ = 1024 bins of equal length (189.5 m). All basal observations within each bin are averaged, leading to a data vector $\bar{B}$ of length 1024. As in [16], we use a wavelet model with two sets of wavelets to provide smoothing. The first group of wavelets captures a smooth signal; the second captures fine-scale or detail signals. Based on the results of [16], we used four smooth and 28 detail wavelets. Specifically, we assume that

$$\begin{array}{c} \bar{B}|\theta \;∼N\left(WC,\sigma_{B}^{2}\;\text{diag}\left\{n_{i}^{-1}\right\}\right), \end{array}$$ (22)

where **W** is the 1024 × 32 matrix of discretized wavelet basis functions; **C** is the 32-dimensional vector of wavelet coefficients; $\sigma_{B}^{2}$ is an error variance; and $\text{diag}\{n_{i}^{-1}\}$ is a 1024 × 1024 diagonal matrix with diagonal elements equal to $n_{i}^{-1}$ where *n*_*i*_ is the number of observations averaged in bin *i* (all *n*_*i*_ are either one or two). We selected Daubechies wavelets, see [17] and [18] for discussion.

**Velocity Model**. We assume that

$$\begin{array}{c} U|\theta ∼N(u,\sigma_{U}^{2}\;I), \end{array}$$ (23)

where **u** is the vector of values given by Eq (18) at the observation locations; $\sigma_{U}^{2}$ is the unknown measurement error variance; and *I* is the identity matrix. Note that the sliding velocity is assumed to be a constant over the study range.

#### Priors for parameters

**Error Variances**. The measurement error variances $\sigma_{S}^{2}$, $\sigma_{B}^{2}$, and $\sigma_{U}^{2}$ were assigned independent, inverse gamma distributions with means and standard deviations (100, 10), (2500, 500), and (9, 3), respectively.

**Surface Model Parameters**. The prior distributions for *β*_0_ and *β*_1_ were specified to be independent normal distributions with large variances: 10,000 for *β*_0_ and 10 for *β*_1_. The means of these normal distributions were set to be equal to the least squares estimates of *β*_0_ and *β*_1_ derived from a traditional analysis fitting the model in Eq (20) to the surface observations.

**Basal Model Parameters**. The prior used for the four coefficients of the smooth-signal wavelets is

$$\begin{array}{c} C_{s}∼N\left(\mu_{s},\sigma_{c}^{2}\;I_{4}\right) \end{array}$$

where ***μ**_s_* is the vector of conventional least squares estimates of Haar-wavelet coefficients. The prior for the remaining 28 coefficients is

$$\begin{array}{c} C_{d}∼N\left(0,\sigma_{d}^{2}\;I_{28}\right). \end{array}$$

We set $\sigma_{c}^{2}=2000$ and $\sigma_{d}^{2}=10000$.

**Velocity Model Parameters**. The prior for the sliding velocity is

$$\begin{array}{c} u_{b}∼N\left(35,14^{2}\right). \end{array}$$

To develop reasonable priors for the Fourier coefficients in Eq (19), we first obtained the least squares fits to the surface and the basal models (20) and (22). These fitted models were substituted into the velocity model (23). We then fitted the result via least squares. As above, the least squares estimates of the Fourier coefficients were used as prior means for the corresponding parameters. These values were on the order of 10^−16^ (which is consistent with the theoretical value of the parameter *A*) except for the frequency parameter *ω* which was estimated to be roughly 10^−5^. These parameters were all assumed to be independent, normal random variables with prior variances equal to 10.

**Fig 3:**
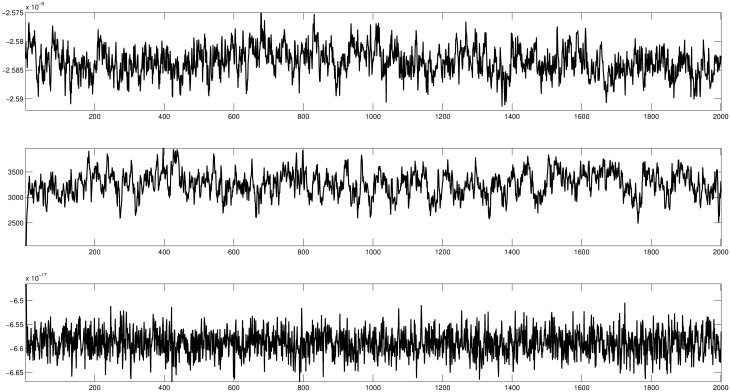
Trace plots for three parameters: Parameter *β*_1_ from the surface model (20)—Top plot; a wavelet coefficient from the basal model—Middle plot and a Fourier coefficient from the flow model (19).

**Fig 4:**
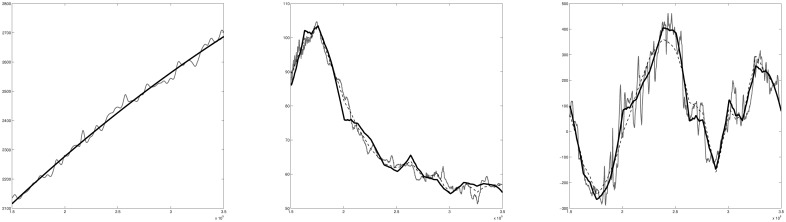
Data (light grey) and posterior means for the diffusion MCMC (solid black lines) and adaptive MCMC (dashed lines). Left panel shows the surface data, middle panel show the velocity data, right panel shows the basal data.

**Performance**. Fig 3 shows trace plots for various parameters. We ran the algorithm for 100000 iterations and thinned it by fifty steps. The diffusion MCMC algorithm performs very well. It appears that it explores the state space properly and mixes very fast. In Fig 4 we show posterior means for the surface, velocity and basal processes. For comparison, we added the posterior means (dashed lines) for the three processes from a much longer adaptive MCMC run. This plot confirms that diffusion MCMC performs as expected, giving similar results to the adaptive MCMC approach with the added benefit of a much shorter computing time.

## Conclusions

Simulation of a diffusion formulated to have stationary distribution coinciding with a target posterior distribution is a viable MCMC method. The approach is comparatively simple to implement since it requires no probability computations such as those needed in Gibbs’ sampling nor any accept-reject steps as in Metropolis algorithms. These advantages can be significant in a variety of settings including mixture likelihoods and/or priors, hierarchical models, nonconjugate priors, and nonlinear models.

The key problem that arises in diffusion MCMC is the approximation of the desired continuous time diffusion by a discrete time Markov chain. Our implementations use Euler discretizations. As reviewed in the Introduction, there are results in the literature providing sufficient conditions under which the discrete approximation has a stationary distribution that approximates that of the target, continuous-time diffusion. Though beyond our scope here, selection of the time-step *h* can be done adaptively, see [6] for some recent theoretical developments in this area.

We implemented diffusion MCMC for a familiar test problem and compared it to an adaptive MCMC procedure. We found that diffusion MCMC out-performed the “state-of-the-art” adaptive MCMC. Next, we implemented the diffusion MCMC approach in a complicated, nonlinear model involving glacial dynamics. Again, we found that our suggested approach performs well, mixing very fast.

In summary, we believe that diffusion MCMC is a valuable addition to the MCMC toolbox. By construction, the DMCMC algorithm has the ability to quickly find important regions of the target distribution, while a classical, even adaptive MCMC, may require longer exploration times (as seen in the glaciological example). It can be applied in great generality and with ease in some complicated contexts for which other MCMC methods are difficult or very time-consuming to implement. DMCMC does carry the baggage of temporal discretization and concern for the quality of the resulting approximation. Nevertheless, the potential power of diffusion MCMC justifies its application and further development.

## Supporting information

S1 Dataset The data set used in this analysis are available in the file S1_Dataset.zip. We provide the surface, basal and velocity data used in this manuscript.
(ZIP)
